# 

**Published:** 1900-09

**Authors:** 


### PERSONALS.

Dr. F. E. Anderson, of Findlay, Ohio, combines the dealing in horses and stable equipments with his professional work.

Dr. Walter L. Burt, now of Portchester, N. Y., has added to his sales farm and stables a training department for the preparation of horses for the show ring.

Dr. Harry Walter, of the Trenton, N. J., Sanitarium, was a visitor to Philadelphia in May. He is now devoting his entire time to human practice in nervous diseases.

President Harger, of the Pennsylvania State Association, is a great favorite among the ladies of the "Quaker City," and when absent from college duties the boys are wont to say that the professor is filling an important mission.

Dr. Charles T. Goentner, of Bryn Mawr, Pa., is a tiller of the soil in conjunction with his veterinary duties, but finds the raising of corn and potatoes a slower method of gaining wealth than waiting professionally on canine and feline pets.

Dr. William Dougherty, of Baltimore, Md., is now the happy possessor of a trotter with a mark along the twenties. It is hard to win a real horse lover away from the trotter.

Dr. W. H. Meadors, of Kansas City, is a British veterinary inspector of horses being purchased in Texas, and is stationed at Denison, Texas.

Dr. T. Earle Budd, of Orange, N. J., is an admirer of the hackneys, and has in his stable Ruby 2d, a royally bred hackney stallion.

Drs. William H. and Bryer Pendry, of Brooklyn, take an active interest in building association affairs, and fill the rôles respectively of president and director in one of the borough's younger associations.

Dr. F. H. Osgood, of Boston, Mass., has been appointed brigade veterinary surgeon of the State militia, and has placed the work on a very high sanitary plane.

Dr. Benjamin D. Pierce, of Springfield, Mass., has gone on a trip to South Africa.

Dr. Reynolds recently presented a paper on "Live-stock Sanitary Work" at the annual meeting in Duluth of the Minnesota State Medical Society.

City Veterinarian Hart, of Philadelphia, finds time now and again to take a dip in the briny waters of the Atlantic Ocean, and when quietly resting on the boardwalk listens to what the wild waves are saying.

Dr. E. D. Hudson, of Gettysburg, Pa., fills the rôle of chairman of the finance committee of the Round Top Democratic Bryan Club.

Drs. D. E. Salmon, of Washington, D. C.; M. P. Ravenel, of Philadelphia; and J. I. Gibson, of Denison, Iowa, are members of the Committee on Animal Diseases and Animal Food of the American Veterinary Medical Association.

Dr. John T. Ferley, Jr., of Philadelphia, has been summoned to defend a $10,000 lawsuit for alienating the affections of one of Philadelphia's ladies.

Dr. W. Horace Hoskins, of Philadelphia, was among those at the college commencement of the New York American Veterinary College.

Dr. A. W. Clement, of Baltimore, Md., and Dr. F. H. Mackie, of Fair Hill, Md., were visitors to the Philadelphia horseshow.

Dr. M. Jacob, house-surgeon of the Veterinary Department of the University of Pennsylvania, recently successfully passed the civil-service examination of the Bureau of Animal Industry and has received instructions to report at New York under Dr. R. W. Hickman.

Dr. E. W. Hammond, of Montreal, Canada, has been acting in place of the Fellow in Bacteriology at the Agricultural College, Guelph, Canada, for several months.

Dr. Frank T. Shannon, of the Bureau of Animal Industry, has been transferred from Washington, D. C., to Seattle, Washington.

Dr. M. S. Lantz, formerly of Du Bois, Pa., has located in Chicago, Ill.

Dr. George Magee, of Uniontown, Pa., was a visitor to Philadelphia in August, incidental to the admission of his son at the Veterinary Department of the University of Pennsylvania.

Professor Andrew Smith is President of the Canadian Horse-breeders' Association. The Association proposes to hold an exposition in 1901 at Toronto.

Dr. Leonard Pearson, of Philadelphia, addressed the Guernsey Breeders' Association at Fulton Station, Lancaster County, in August.

Dr. John J. Repp, of Ames, Iowa, has an excellent article on the "Dangers of Meat and Milk when Animals are Tuberculous," in the *Philadelphia Medical Journal.*

The success of the Alumni of the College of Veterinary Medicine, Ohio State University, is again brought to notice by the recent appointments to the Government meat-inspection service of Dr. R. N. Mead, Grand Rapids, Ohio; Dr. W. F. Lavery, South Salem, Ohio; and Dr. H. J. Hammond, Akron, Ohio. Up to date, 48 per cent. of the graduates of this college hold official positions.

Dr. T. F. Moyle, of Waterford, Wis., will unite with Dr. W. E. A. Wyman, of the JOURNAL staff of collaborators, in the preparation of a special contribution on "Surgical Obstetrics" for a future number of the JOURNAL.

Dr. Wm. H. Wray, of London, Eng., is assisting our Government in the introduction of sweet potatoes in England and other European countries.

Dr. Wm. R. Andress, of Philadelphia, Pa., graduate of Veterinary Department University of Pennsylvania, class of 1900, has been appointed by the Government as meat-inspector at Kansas City, Kan.

Dr. Richard F. Powers, now of Portland, Ore., has just returned from Manila, P. I., where he held the position of chief veterinarian of the Department of Health. Dr. Powers returns to the United States on account of ill-health.

Dr. Wm. H. Lowe, of Paterson, N. J., Treasurer of the American Veterinary Medical Association, is enthusiastic over the project of taking the meeting to Atlantic City, N. J., in 1901.

Dr. E. H. Nodyne, of Sodus, N. Y., is manager of the Sodus Coach-horse Company.

Dr. J. C. McNeil, of Pittsburg, Pa., was among the boardwalk strollers at Atlantic City, N. J., early in September.

That veteran Association member, Dr. Wm. Dougherty, of Baltimore, Md., says the Detroit convention was the first shirt-waist meeting of the Association he had ever attended.

Veterinary Surgeon Desmond, formerly of Warnambool, is now at Adelaide, South Australia, and represents the Government Department as veterinary surgeon and chief inspector of cattle to the central board of health. Dr. Desmond was for a period bacteriologist at the Melbourne Veterinary College.

Dr. Edgar W. Powell, of Newtown Square, class of 1900, Veterinary Department University of Pennsylvania, has located at Oxford, Pa., succeeding Dr. McClure at the latter point.

Dr. E. B Ackerman and wife, of Brooklyn, N. Y., visited Niagara Falls on their way to the Detroit Convention of the A merican Veterinary Medical Association.

Dr. M. E. Knowles, of Helena, Montana, and State Veterinarian for his adopted commonwealth, will make battle for the mayoralty of Helena under the Democratic flag.

Dr. R. R. Bell, of Brooklyn, N. Y., continues to make permanent investments in fair Virginia, where he recently added sixty acres to his rural estate.

Dr. Geo. White, of Nashville, Tenn., was a visitor to Detroit in the interest of his city, looking to better legislation regulating milk- and meat-inspection. He considers the Zenoleum sheep dip a very potent remedy.

Dr. Jas. L. Robertson, of New York City, spent several days at the farm of his friend, Dr. Eugene Burget, in Ohio, prior to their attendance at the annual convention of the A. V. M. A., in Detroit.

Dr. R. H. Harrison, of Milwaukee, Wis., is the proud possessor of his first son, and rumor hath it that this event caused his absence from Detroit.

Dr. J. C. Lacock, of Allegheny, Pa., finds the quietude of Ocean City, N. J., the place *par excellence* for a rest from the exactions of an active practice in the Smoky City.

Dr. H. F. Palmer, of Detroit, Mich., fills the rôle of veterinarian to the drug firm of Parke, Davis & Co.

Dr. T. B. Rogers, of Woodbury, N. J., is preparing a veterinary medical digest for the well-known firm of Messrs. H. K. Mulford Co., of Philadelphia.

Dr. P. F. Bahnsen, formerly of Fort Valley, Ga., has now located at Americus, Ga.

Dr. D. E. Kimmell, veterinarian and proprietor of St. James Hotel, Corry, Pa., will shortly remove to East Palestine, O., where he will enter into the practice of the veterinary science.

Dr. J. M. Carter, formerly of Chatham, Pa., has located in Philadelphia.

Dr. M. S. Lantz, of the Bureau of Animal Industry, has been transferred from the Union Stock Yards, Chicago, to the Quarantine Station at Pendleton, Oregon, where his special duties will be the looking after sheep "scabies."

Dr. Leonard Pearson, State Veterinarian, spent several days in Western Pennsylvania in September, investigating several reported outbreaks of rabies.

Dr. A. L. Parker has resumed practice at Providence, R. I., after an absence of one year spent at Riverdale, N. H., for the benefit of his health.

Dr. H. D. Gill, with his fast trotting mare "Rosalie," won the silver cup trophy at the Queen's County Fair in September. "Rosalie" is one of the very fastest pacemakers on the "Speedway."

Drs. Thomas B. Rayner, of Chestnut Hill; W. L. Rhoads, of Lansdowne, and W. Horace Hoskins, of Philadelphia, were present as delegates or visitors to the Veterinary Medical Association of New Jersey at the annual meeting at Atlantic City in September.

Dr. Coleman Nockolds, of the Grand Rapids Veterinary College, who successfully passed the army service examination, was assigned to the 1st U. S. Cavalry, and has gone to Manila, P. I.

Dr. Frank Standen, of Philadelphia, has returned from his European tour much improved in health.

Dr. A. T. Sellers will resume practice in Camden, N. J., early in October.

Dr. P. K. Jones, of Pittsburg, Pa., takes an active part in urging army legislation, and finds an avenue in the public press to assist the cause.

Dr. J. W. Petty, of Winston, N. C., has been on the sick-list for several months, but is now convalescent.

